# The English National Cohort Study of Flooding & Health: psychological morbidity at three years of follow up

**DOI:** 10.1186/s12889-020-8424-3

**Published:** 2020-03-30

**Authors:** Ranya Mulchandani, Ben Armstrong, Charles R. Beck, Thomas David Waite, Richard Amlôt, Sari Kovats, Giovanni Leonardi, G. James Rubin, Isabel Oliver

**Affiliations:** 1 Field Epidemiology, Field Service, National Infection Service, Public Health England, Bristol, BS1 6EH UK; 2 NIHR Health Protection Research Unit in Evaluation of Interventions, University of Bristol, Bristol, BS8 2BN UK; 3 NIHR Health Protection Research Unit in Environmental Change and Health at the London School of Hygiene and Tropical Medicine, London, WC1H 9SH UK; 4 Population Health Sciences, Bristol Medical School, University of Bristol, Bristol, BS8 2PS UK; 5 Global Public Health, Field Service, Public Health England, Wellington House, London, SE1 8UG UK; 6 Centre for Radiation, Chemicals and Environmental Hazards, Public Health England, Chilton, Didcot, Oxfordshire OX11 0RQ UK; 7 NIHR Health Protection Research Unit in Emergency Preparedness and Response at King’s College London, London, UK

**Keywords:** Post-traumatic stress disorder, Depression, Anxiety, Psychological morbidity, Flooding

## Abstract

**Background:**

Flooding is expected to increase due to climate change, population growth and urban development. The longer-term mental health impacts of flooding are not well understood. In 2015, the English National Study of Flooding and Health was established to improve understanding of the impact of flooding on health and inform future public health action.

**Methods:**

We used 3 years of data from the English National Study of Flooding and Health. Participants who had consented to follow up were sent a questionnaire. Participants were classified into either “unaffected”, “disrupted” or “flooded” according to their exposure. Logistic regression models were used to calculate adjusted odds ratios for probable depression, anxiety and post-traumatic stress disorder (PTSD) in each exposure group. The Wald test was used to assess the difference in probable mental health outcomes for those who did and did not experience “persistent damage” to their home. Conditional logistic regression was conducted to assess change in prevalence over the 3 years and to identify possible determinants of recovery.

**Results:**

Eight hundred nineteen individuals were included in the final analysis – 119 were classified as unaffected, 421 disrupted and 279 flooded. Overall, 5.7% had probable depression, 8.1% had probable anxiety and 11.8% had probable PTSD, with higher prevalence in the flooded group compared with the unaffected group. After adjustment for potential confounders, probable mental health outcomes were higher in the flooded group compared to the unaffected group, significantly for probable depression (aOR 8.48, 95% CI 1.04–68.97) and PTSD (aOR 7.74, 95% CI 2.24–26.79). Seventy-seven (9.4%) participants reported experiencing persistent damage to their home, most commonly damp (*n* = 40) and visible mould (*n* = 26) in liveable rooms. Of the 569 participants who responded at all 3 years, a significant reduction in prevalence for all probable mental health outcomes was observed in the flooded group.

**Conclusions:**

Flooding can have severe long-lasting consequences on mental health in affected populations. If these problems are not identified and treated early, they may persist for years. Further research is necessary to develop and evaluate interventions to increase resilience in at risk populations and to ensure timely access to support services following flooding.

## Background

Flooding is the most common natural disaster worldwide and has been shown to have an adverse impact on both physical and psychological health [1]. In England, it is estimated that around 5.2 million properties are at risk of flooding [2]. Frequency and intensity of floods are anticipated to increase in the future due to population growth, urban development on flood plains, and climate change [3, 4].

In high-income countries such as the UK, the greatest burden of disease following flooding is adverse mental health outcomes [5]. In addition, displacement from homes can result in stress arising from dealing with household repairs and disruption to public services [6]. A number of factors have been found to increase vulnerability to experiencing psychological impacts following extreme weather, including older age, pre-existing medical conditions, inadequate insurance cover and social deprivation [7].

There is a paucity of studies quantifying the longer-term impacts of flooding on health, particularly beyond the first year post-flooding [8]. Following floods affecting England in 2013–2014, Public Health England (PHE) established the National Study of Flooding and Health (NSFH), to investigate the long-term impact of flooding and associated disruption on psychological health. The study aims to support preparedness and response activities to future flooding events.

The NSFH has previously identified a significant adverse impact on mental health, both at one and 2 years post-flooding, in those whose homes were flooded and whose lives were otherwise disrupted by flooding, compared with those unaffected [9, 10]. The NSFH also identified that adverse outcomes are associated with secondary stressors [11], such as insurance-related issues [12], and with displacement from home without warning [13]. In this study, we aim to assess mental health morbidity at 3 years post-flooding and the impact of persistent flood-related damage in the home. We also aim to assess the prevalence change over the three-year period, to identify possible predictors for psychological recovery.

## Methods

### Study design

This study is at year three of follow-up as part of the English NSFH, which was designed as a longitudinal observational open cohort. The participants are people affected by flooding between 1 December 2013 and 31 March 2014 (which are described in more detail elsewhere [9]).

### Study population

The original cohort comprised of 2126 participants, with 1408 providing consent for follow-up [9]. At year two, of the 1408 contacted a total of 1064 responded [10]. 1361 participants were contacted at year three. This included all participants who had consented to follow-up at year one, irrespective of their response at year two, had not withdrawn consent subsequently and remained contactable.

### Data collection

Participants were sent a 21-item bespoke questionnaire by either post or email. At year three, the questionnaire collected the following demographic information: marital status, educational level, employment status and presence of ongoing illness. Participant sex, ethnicity and age were collected at year one.

The participants had been classified in year one according to their exposure to flooding in the winter of 2013/14. The categories were either “unaffected”, “disrupted” (life disrupted by flooding, but no entry of water into any liveable room of the home) or “flooded” (entry of water into at least one liveable room of the home).

The questionnaire included validated instruments to determine probable psychological outcomes based on self-reported symptoms. The instruments used were the Patient Health Questionnaire (PHQ-2) for depression [14], Generalized Anxiety Disorder Scale (GAD-2) for anxiety [15] and PTSD checklist (PCL-6) for PTSD [16]. Cut-off scores were ≥ 3 for PHQ-2 and GAD-2 and ≥ 14 for PCL-6, respectively.

The questionnaire also included questions to determine whether the participant’s home had ongoing damage from the original floods (“persistent damage”), whether they had experienced any new episodes of flooding, status of any insurance re-payment and other potential secondary stressors (dealing with insurance-related issues, dealing with home repairs, concerns about own health, relationship problems, disagreements with neighbours and concerns about the value of the home).

“Persistent damage” was defined as ongoing flood-related issues in the home damp in liveable rooms, visible mould in liveable rooms, problems with damp or water in non-liveable rooms, sewage backing up, problems with septic tank and problems with other utilities (drinking water, gas, oil, electricity etc) attributed to the floods in the winter of 2013/2014.

### Statistical analysis

We performed a descriptive analysis of the sociodemographic characteristics of respondents, their exposure to flooding and any experience of persistent damage, and mental health outcomes of probable depression, anxiety and PTSD.

It is important to note that the crude mental health prevalence presented are not exactly comparable to those presented at year two of this study, in the previously published paper by *Jermacane* et al. [10]. In *Jermacane* et al*, 2018*, individuals who had responded to some, but not all mental health questions, were included in the denominator data, but in the present study those subjects were excluded, in line with the approach of *Waite* et al. [9] for year one data. In our paper, we have calculated prevalence according to the method used by *Waite* et al*, 2017* at all 3 years, to allow for easier comparison across all 3 years.

Crude logistic regression models were run for all exposure groups to test for associations between exposure variables (flooding and disruption from flooding) and probable mental health outcomes, using those unaffected as the reference group.

Multivariable logistic regression models were run to adjust for a priori potential confounders, including age group, sex, ethnic group, pre-existing illness, deprivation score (Index of Multiple Deprivation, IMD), marital status, education and employment.

We used the Wald test to assess whether there was a significant difference in probable mental health outcomes between those who experienced persistent damage at year three and those who did not; for this analysis we only included disrupted and flooded respondents, with the disrupted group as the reference. Conditional logistic regression was conducted to test for significant changes in prevalence over the 3 years by each exposure group and to identify possible determinants of recovery for mental health outcomes. Only those who responded in all 3 years were included in the matched analyses. All data were merged, cleaned and analysed in R software version 3.5.0 (R Foundation for Statistical Computing, Vienna, Austria).

## Results

Of the 1361 participants contacted at year three, 896 responded with a questionnaire, however 29 questionnaires were blank and were not included in the analysis (63.7% valid response rate). A further 48 exclusions were made, including 9 duplicates, 3 who reported a new episode of flooding and 36 who had a missing exposure status at year one. Of the 819 included in the final analysis, 119 (14.5%) were classified as unaffected, 421 (51.4%) as disrupted and 279 (34.0%) as flooded. Of those, 569 had completed the questionnaire in all 3 years, with 93 (16.3%) classified as unaffected, 289 (50.8%) as disrupted and 187 (32.9%) as flooded.

Overall, approximately 5.7% reported symptoms of probable depression, 8.1% of probable anxiety and 11.8% of probable PTSD, with the prevalence of all adverse probable mental health outcomes higher in the flooded group than unaffected (Table 1).

**Table 1:** Crude prevalence of mental health outcomes by exposure group (year 3)

Outcome	Overall cohort	Exposure group		
		Unaffected	Disrupted	Flooded
Probable depression	42/733 (5.7%)	1/112 (0.9%)	22/380 (5.8%)	19/241 (7.9%)
Probable anxiety	59/731 (8.1%)	4/114 (3.5%)	27/378 (7.1%)	28/239 (11.7%)
Probable PTSD	91/771 (11.8%)	3/117 (2.6%)	43/397 (10.2%)	45/257 (17.5%)

The adjusted odds ratio (aOR) of probable depression and PTSD were significantly higher in the flooded group compared with the unaffected group, with aOR 8.48 (95% CI 1.04–68.97) and aOR 7.74 (95% CI 2.24–26.79), respectively. The aOR of probable anxiety was elevated in the flooded group, compared with the unaffected group but not significantly (aOR 2.68, 95% CI 0.88–8.20). Participants who were disrupted by flooding had increased odds of PTSD (aOR 4.33, 95% CI 1.26–14.92), compared with the unaffected group; no other probable mental health outcomes in the disrupted group were statistically significant (Table 2).

**Table 2:** Crude and adjusted odds ratios (aOR) of mental health outcomes by exposure group

Outcome by exposure	Prevalence	Crude OR (95% CI)	aOR^a^ (95%CI)	*P* value
Probable depression				
Unaffected	0.9%	ref	ref	
Disrupted	5.8%	6.82 (0.91–51.18)	5.89 (0.74–47.10)	0.094
Flooded	7.9%	9.50 (1.25–71.88)	8.48 (1.04–68.97)	0.046
Probable anxiety				
Unaffected	3.5%	ref	ref	
Disrupted	7.1%	2.12 (0.72–6.17)	1.59 (0.52–4.83)	0.412
Flooded	11.7%	3.65 (1.24–10.7)	2.68 (0.88–8.20)	0.084
Probable PTSD				
Unaffected	2.6%	ref	ref	
Disrupted	10.8%	4.62 (1.40–15.16)	4.33 (1.26–14.92)	0.020
Flooded	17.5%	8.07 (2.45–26.53)	7.74 (2.24–26.79)	0.001

^a^Adjusted odds ratios are adjusted for age, sex, pre-existing illness, deprivation score, marital status and education and employment

Seventy-seven (9.4%) participants reported persistent damage to their home because of the original flooding episode. The most commonly reported issues were damp in liveable rooms (*n* = 40), visible mould in liveable rooms (*n* = 26), problems with damp or water in non-liveable rooms such as garage, cellar or basement (*n* = 12) and drains backing up and flooding (*n* = 10). After adjusting for potential confounders, those who reported persistent home damage at year three were statistically more likely to suffer from depression and PTSD, compared with those who did not report persistent damage, however anxiety was not significantly elevated in this group (Table 3).

**Table 3:** Crude and adjusted odds ratios (aOR) of mental health outcomes of participants with and without persistent damage to year three

Outcome by exposure	Prevalence (n/N)	Crude OR (95% CI)	aOR^a^ (95%CI)	*P* value
Probable depression				
Disrupted				
No persistent damage	4.8% (17/356)	ref	ref	
Persistent damage	20.8% (5/24)	5.20 (1.73–15.61)	19.30 (3.99–93.24)	< 0.001
Flooded				
No persistent damage	5.6% (11/197)	ref	ref	
Persistent damage	18.1% (8/44)	3.68 (1.38–9.78)	6.02 (1.61–22.5)	0.008
Probable anxiety				
Disrupted				
No persistent damage	6.5% (23/354)	ref	ref	
Persistent damage	16.7% (4/24)	2.85 (0.90–9.04)	5.53 (1.31–23.30)	0.019
Flooded				
No persistent damage	10.4% (20/196)	ref	ref	
Persistent damage	18.6% (8/43)	1.97 (0.80–4.82)	1.92 (0.67–5.54)	0.227
Probable PTSD				
Disrupted				
No persistent damage	9.7% (36/371)	ref	ref	
Persistent damage	24.0% (6/25)	2.91 (1.09–7.76)	3.85 (1.13–13.11)	0.031
Flooded				
No persistent damage	13.7% (29/211)	ref	ref	
Persistent damage	30.2% (13/43)	2.70 (1.26–5.78)	4.56 (1.73–11.99)	0.002

^a^Adjusted odds ratios are adjusted for age, sex, pre-existing illness, deprivation score, marital status and education and employment

**Fig. 1:**
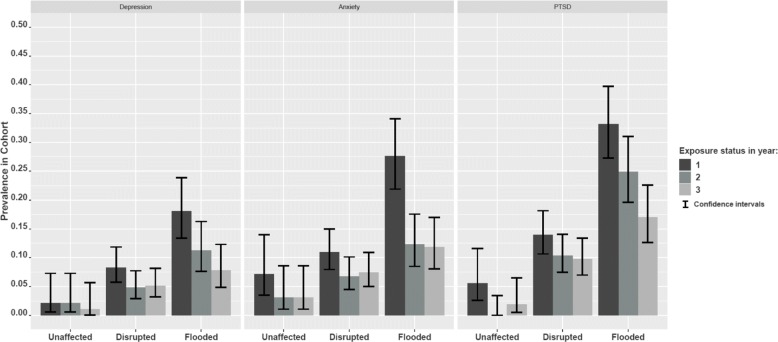
Prevalence of mental health outcomes by exposure group over 3 years post-flooding of participants who responded all 3 years

We observed a prevalence change of probable mental health outcomes over three consecutive years post-flooding, for the 569 participants who completed the questionnaire in all 3 years (Fig. 1, Supplementary Table 1). In the flooded group, we observed a significant reduction in prevalence across all three probable mental health outcomes: depression (year one 20.8%, year two 11.2%, year three 7.8%, *p* = 0.0014), anxiety (year one 27.6%, year two 12.3%, year three 11.8%, *p* < 0.001) and PTSD (year one 33.2%, year two 24.9%, year three 17.1%, *p* = 0.001). The reduction was suggestive in the disrupted group for depression (year one 8.3%, year two 4.8%, year three 5.1%, *p* = 0.05) and in the unaffected group for PTSD (year one 5.6%, year two 0%, year three 1.9%, *p* = 0.045), but not for anxiety in either the disrupted or unaffected group. No significant predictors were identified for the reductions in prevalence of adverse mental health outcomes.

## Discussion

Few studies have focused on the long-term prevalence of mental health problems in those affected by flooding, with systematic mapping reviews by *Zhong* et al*, 2018* and *Fernandez* et al*, 2015* highlighting the lack of studies conducted on this topic two or more years post-flooding [1, 17]. Our paper assessed the prevalence of probable mental health outcomes 3 years after a flooding event.

We identified that the adverse impact of flooding on mental health persists for at least 3 years after the flooding event, with a higher prevalence of psychological morbidity (significantly for depression and PTSD) in flooded participants, compared with those unaffected. Many individuals reported persistent damage to their homes, which was a strong predictor for poorer mental health outcomes, compared with other people who were exposed (disrupted or flooded) but who did not report experiencing persistent damage issues.

Overall the data show a reduction in psychological morbidity over the 3 years in the flooded group, a suggestive decline in the disrupted group and no significant differences in the unaffected group. Unfortunately, we were unable to identify any predicators of this recovery in this cohort; previous studies has found factors such as availability of social support and personal coping style could influence recovery from PTSD post-flooding [18, 19]; however, these were conducted in China and more research is required on understanding the mechanisms for recovery in other contexts post-flooding.

We observed nearly half the prevalence of PTSD symptoms in the flooded group at year three post-flooding, compared with year one. This is in line with previous studies on PTSD related to natural disasters where one meta-analysis calculated a spontaneous remission rate of 60.0% [20], and may partly reflect the resolution of ongoing stressors that were helping to maintain distress in this group.

Within our study we observed 17.5% of flooded individuals with scores that indicated probable PTSD at 3 years after flooding, which is line with previous studies – 22% of individuals in South Korea at 18 months after flooding [21] and 8.6% of individuals 2.5 years after flooding in China [22] experienced probable PTSD. However, despite the decline in prevalence observed in people who have experienced flooding over the 3 years, there is still persistence of psychological morbidity, which may indicate a possible risk of chronic mental health problems if affected people do not receive suitable treatment.

### Limitations

There are several limitations with our study. There were a low number of cases in the unaffected group, which impacts the precision and power of our study – particularly for probable depression in the unaffected group, which is only based on one case.

Our study was conducted in response to flooding that occurred in 2013–2014 in the south of England within a homogenous population in terms of income, age and ethnic group; it may not generalizable for all English populations or representative across other geographical contexts. As we excluded people who reported experiencing a further episode of flooding (since the original floods in 2013–2014), our data does not consider the impact of repeated flooding on the extent of mental health outcomes.

A strength of our paper is the use of conditional logistic regression, where we have matched the same individuals over the 3 years. This allowed us to understand the change in mental health prevalence over time without having to control for potential confounders. This is particularly important, as the review by *Fernandez* et al*, 2015* identified that most other studies had not taken confounding into account, limiting overall confidence in their study conclusions [1].

### Further research

We have identified that experience of flooding followed by persistent damage to the home is a significant predictor for poorer mental health outcomes. It would be important to understand in more detail the types of damage experienced and how these impact on mental health, particularly in vulnerable groups who may experience and respond to damage differently, to guide appropriate public health action. Studies are also needed to develop and evaluate interventions, such as social support, to reduce the impact of flooding on mental health.

## Conclusions

This study has shown that the adverse impact of flooding on mental health persists for at least 3 years after the event, and that persistent damage to liveable rooms in the home is associated with more severe mental health outcomes. Work is needed to develop and evaluate interventions to increase resilience within populations at risk of flooding and to ensure prompt access to appropriate services following a flooding event.

## Supplementary information

**Additional file 1: Supplementary Table 1.** Prevalence of mental health outcomes by exposure group over 3 years post-flooding of participants who responded at all 3 years.

## Data Availability

The datasets used and analysed during the study are available from Public Health England on reasonable request.

## Abbreviations

aOR: Adjusted odds ratio
CI: Confidence interval
NSFH: National Study of Flooding and Health
OR: Odds ratio
PHE: Public Health England
PTSD: Post-traumatic stress disorder
UK: United Kingdom
