# Supplementary material for: The English National Cohort Study of Flooding & Health: psychological morbidity at three years of follow up
Source: BMC Public Health. 2020 Mar 30;20:321. doi: 10.1186/s12889-020-8424-3 (PMC7104503; doi:10.1186/s12889-020-8424-3)
Supplement: Supplementary file 1 — Additional file 1: Supplementary Table 1. Prevalence of mental health outcomes by exposure group over 3 years post-flooding of participants who responded at all 3 years. [file 12889_2020_8424_MOESM1_ESM.docx]

**Additional file 1**

**Supplementary Table 1** Prevalence of mental health outcomes by exposure group over three years post-flooding of participants who responded at all three years

| Outcome | Overall cohort | Exposure group | | |
| --- | --- | --- | --- | --- |
|  |  | Unaffected | Disrupted | Flooded |
| *Probable depression* | | | | |
| Year 1 | 65/615 (10.6%) | 2/96 (2.1%) | 26/314 (8.3%) | 37/205 (20.8%) |
| Year 2 | 40/615 (6.5%) | 2/96 (2.1%) | 15/314 (4.8%) | 23/205 (11.2%) |
| Year 3 | 33/615 (5.4%) | 1/96 (1.0%) | 16/314 (5.1%) | 16/205 (7.8%) |
| *Probable anxiety* | | | | |
| Year 1 | 97/611 (15.9%) | 7/98 (7.1%) | 34/310 (11.0%) | 56/203 (27.6%) |
| Year 2 | 49/611 (8.0%) | 3/98 (3.1%) | 21/310 (6.7%) | 25/203 (12.3%) |
| Year 3 | 50/611 (8.2%) | 3/98 (3.1%) | 23/310 (7.4%) | 24/203 (11.8%) |
| *Probable PTSD* | | | | |
| Year 1 | 124/654 (19.0%) | 6/108 (5.6%) | 46/329 (14.0%) | 72/217 (33.2%) |
| Year 2 | 88/654 (13.5%) | 0/108 (0%) | 34/329 (10.3%) | 54/217 (24.9%) |
| Year 3 | 71/654 (10.9%) | 2/108 (1.9%) | 32/329 (9.7%) | 37/217 (17.1%) |
